# Drug utilization pattern among pregnant women attending maternal and child health clinic of tertiary hospital in eastern Ethiopia: Consideration of toxicological perspectives

**DOI:** 10.1186/s13104-018-3966-5

**Published:** 2018-12-04

**Authors:** Neim Bedewi, Mekonnen Sisay, Dumessa Edessa

**Affiliations:** 1 School of Pharmacy, College of Health and Medical Sciences, Haramaya University, P.O.Box, 235, Harar, Ethiopia; 2 Department of Pharmacology and Toxicology, School of Pharmacy, College of Health and Medical Sciences, Haramaya University, P.O.Box, 235, Harar, Ethiopia; 3 Department of Pharmacy Practice, School of Pharmacy, College of Health and Medical Sciences, Haramaya University, P.O. Box, 235, Harar, Ethiopia

**Keywords:** Drug utilization, Pregnancy, Tertiary hospital

## Abstract

**Objective:**

This study is aimed to investigate drug utilization pattern among pregnant women attending maternal and child health clinic of tertiary hospital in eastern Ethiopia from March 1 to April 20, 2018.

**Result:**

A total of 369 pregnant women medical records were reviewed. The mean age of pregnant women was 24.34 (± 4.48) years and the majority of them were within the age of 18-25 years. About three-fourths (n = 277, 75.1%) of them were urban residents. Besides, 314 (85.1%) women had taken at least one drug with a total of 377 drugs prescribed. From which, supplemental drugs accounted majority of the drug therapy (84.88%) whereas non-supplemental drugs (15.12%) were used by 41 pregnant women during the review period. According to Food and Drug Administration FDA pregnancy risk classification, 320 (84.88%) drugs were prescribed from category A; 33 (8.75%) drugs were from category B; 19 (5.04%) drugs were from category C and 5 (1.33%) drugs were from category D. There was no drug prescribed from category X. As this result indicated, there is a decrease in the prevalence of drug use from Category A to X as the possibility of potential risk to fetus might outweigh the potential benefit to the mother. Some drugs were utilized from category D for treatment of chronic illnesses.

**Electronic supplementary material:**

The online version of this article (10.1186/s13104-018-3966-5) contains supplementary material, which is available to authorized users.

## Introduction

Despite the lack of adequate studies on safety of prescription drugs for pregnant women, available evidence has shown that healthcare professionals prescribe and pregnant women take a surprisingly large number of drugs. Furthermore, 86% of the women had taken at least one prescription medication during their pregnancies. Despite several physiological, pharmacokinetic and pharmacodynamic changes occurring during pregnancy, pregnant women are still considered therapeutic orphans, since the majority of available drugs were not adequately studied in pregnancy [1–4].

Pregnant women have been often excluded from clinical trials and evidences generated from animal-based studies are not often suitable for extrapolation to indicate teratogenicity in humans. Hence, drug use by pregnant women is considered experimental in most clinical practices. However, the use of medications is sometimes mandatory in the treatment of women of reproductive age, breast feeding and during pregnancy [5, 6]. Healthcare professionals should ensure that optimal medications are prescribed when treating women of childbearing potential with chronic diseases [7].

A large number of studies have been conducted in developing and developed countries. Most of them have reported the utilization of large number of drugs during pregnancy with substantial number of drugs from category D and X of Food and Drug administration (FDA) pregnancy risk categories [8]. Although it seems rare, previous studies demonstrated that the use of drugs from FDA pregnancy risk category X had been observed during pregnancy [9–12]. This study is, therefore, designed to investigate the drug utilization pattern and toxicological perspectives among pregnant women attending tertiary hospital in eastern Ethiopia.

## Main texts

### Methods

#### Study area, design and period

The study was conducted at Hiwot Fana Specialized University Hospital (HFSUH), Harar which is located 526 km away from Addis Ababa to the east. There are three governmental hospitals in Harar town and HFSUH was purposely selected as it is a tertiary care teaching hospital of Haramaya University and hosts majority of patient attendees from Harar city and its surrounding. An institution-based cross-sectional study was conducted from March 1 to April 20, 2018 on one year medical records (from September 1, 2016 to August 31, 2017) of pregnant women attending maternal and child health (MCH) clinic of the hospital.

#### Study population

All medical records of pregnant women who attended MCH clinic of HFSUH from Sep 1, 2016 to Aug 31, 2017 were considered as study population from which sampling units were drawn.

#### Sample size determination and sampling techniques

The sample size determination was performed according to single population proportion formula as shown hereunder

$${\text{n }} = \frac{{z^{2} p\left( {1 - p} \right)}}{{d^{2} }} = \frac{{\left( {1.96} \right)^{2} *0.875\left( {1 - 0.875} \right)}}{{\left( {0.03} \right)^{2} }} = { 466}. 8 6 \sim 4 6 7$$

where, p = prevalence/proportion of drug use during pregnancy, 87.5% [8], d = margin of error which is 3%, z = confidence level at 95% = 1.96. The size of study population per year (the total number of pregnant women who had follow up during the review period) was found to be 1410 (< 10,000). Thus, adjustment had been performed to get the final sample size

$$\varvec{n}_{{\varvec{f }}} = \varvec{ }\frac{{\varvec{ni}}}{{1 + \frac{{\varvec{ni}}}{\varvec{N}}}} = \frac{467}{{1 + \frac{467}{1410}}}\varvec{ } = \varvec{ }351$$

where; $\varvec{n}_{{\varvec{f }}}$ = final sample size; ni = initial sample size; N = Study population. Up on adding 5% methodological contingency, the final sample size was found to be 369. Simple random sampling was applied to obtain sampling units (medical records).

#### Data collection processes

Data abstraction format containing socio-demographics (Age, gravidity and resident), clinical and drug related variables (Trimester of pregnancy, supplemental and other drugs utilization and medical conditions diagnosed during pregnancy), FDA pregnancy risk categories and prevalence of components of dosage regimen was utilized to extract important information from medical records.

#### Pretest and data quality control

Pretest was conducted in Jugel hospital, found in the same town, for the sake of amending the data collection tool to best fit the existing context. Data collecting format was also cross-matched with available information on records. Data cleaning was performed on daily basis. Incomplete charts were discarded.

#### Data processing and analysis

The data were coded, entered and analyzed by using Statistical Package for Social Sciences (SPSS) version 20 (IBM statistics, Armonk, NY, USA). Data were presented in tables and figure.

### Results

#### Socio-demographic characteristics

A total of 369 pregnant women medical records were reviewed for this study. From these, the larger proportion of women 220 (59.62%) were found multigravida. The mean age of pregnant women was 24.34 (± 4.48) years and the majority of them were within the age of 18-25 years. About three-fourths (n = 277, 75.1%) of them were urban residents (Table 1).

**Table 1:** Socio-demographic characteristics of pregnant women attending MCH clinic of HFSUH, eastern Ethiopia

Variables and category	Frequency (%)
Age (years)	
< 18	31 (8.4)
18–25	201 (54.5)
26–30	107 (29.0)
> 30	30 (8.1)
Gravidity	
Primigravida	149 (40.4)
Multigravida	220 (59.6)
Area of resident	
Urban	277 (75.1)
Rural	92 (24.9)

#### Clinical and drug use characteristics

From the total of 369 pregnant women, 314 (85.1%) had taken at least one drug. A total of 377 drugs were prescribed for such pregnant women, from which supplemental drugs accounted majority of the drug therapy (n = 320, 84.88%) whereas non-supplemental drugs (n = 57, 15.12%) were used by 41 pregnant women during the review period. Among supplemental drugs, iron/folate combinations were found predominately prescribed medications (n = 313; 97.81%) with the highest consumption seen in the 1st and 2nd trimester. The average number of drugs prescribed per pregnant women was found to be 1.21. From non-supplemental drugs, antibiotics were the most commonly prescribed drugs followed by gastrointestinal drugs. From pregnant women prescribed with non-supplemental drugs, there was no recorded diagnosis on seven pregnant women medical records; 29 women had been diagnosed with acute illnesses (by considering three pregnant women with two diagnoses each). From acute illnesses, dyspepsia and urinary tract infection took the largest percentage share. Five pregnant women had been diagnosed with chronic illnesses (Table 2). Excluding iron/folate, 39, 14 and 11 drugs were used during the first, second and third trimester of pregnancy, respectively (Table 3).

**Table 2:** Frequency distribution of iron/folate used across trimesters, drug category utilized and medical conditions diagnosed among pregnant women attending MCH clinic of HFSUH, eastern Ethiopia from September 1, 2016 to August 31, 2017

Clinical and drug related variables	Frequency (%)
Iron/folate use by Trimester of pregnancy	
1st	99 (31.63)
2nd	74 (23.64)
3rd	44 (14.06)
1st and 2nd	32 (10.22)
1st and 3rd	16 (5.11)
2nd and 3rd	25 (7.99)
Throughout the gestation	23 (7.35)
Total	313 (100)
Drug category used during pregnancy	
Antibiotics	22 (5.3)
Gastro-intestinal drugs	17 (4.51)
Analgesics	9 (2.39)
Vitamins and minerals (supplemental)	320 (84.88)
Others^a^	9 (2.92)
Medical condition diagnosed in pregnant women	
Acute illnesses	29 (70.73)
Amebiasis	2 (6.9)
Common cold	1 (3.45)
Dyspepsia	9 (31.03)
*H. pylori* infection	1 (3.45)
Hyper-emesis (morning sickness)	5 (17.24)
Pneumonia	5 (17.24)
Urinary tract infections	6 (20.69)
Chronic illnesses	5 (12.19)
Asthma	2 (40.0)
Bipolar without psychotic feature	1 (20.0)
Epilepsy	1 (20.0)
HIV/AIDS	1 (20.0)

^a^Respiratory drugs, anti-convulsant, anti-psychotic, corticosteroids

**Table 3:** Medications used by pregnant women at different trimesters and their US FDA pregnancy risk classification in HFSUH, eastern Ethiopia from September 1, 2016 to August 31, 2017

Drug name	1st trimester Frequency	2nd trimester Frequency	3rd trimester Frequency	Total Frequency	FDA risk category
Amoxicillin	3	4	0	7	B
Augmentin	1	0	0	1	B
Azithromycin	4	1	0	5	B
Carbamazepine	2	0	0	2	D
Ceftriaxone	1	0	0	1	B
Cephalexin	1	1	1	3	B
Cimetidine	2	1	2	5	B
Cotrimoxazole	1	0	0	1	D
Chlorpromazine	4	0	0	4	C
Diazepam	1	0	0	1	D
Diclofenac	3	0	0	3	C
Diphenhydramine	0	1	0	1	B
Haloperidol	1	0	0	1	C
Hydrocortisone	1	0	0	1	C
Iron/folate^a^	✓	✓	✓	313	A
Mebendazole	1	0	0	1	C
Metoclopramide	1	1	0	2	B
Metronidazole	0	1	1	2	B
MTS	1	0	1	2	C
Multi-vitamin^a^	4	1	2	7	A
Omeprazole	1	1	1	3	C
Paracetamol	2	2	0	4	B
Prednisolone	1	0	0	1	D
Salbutamol	1	0	1	2	C
TDF + 3TC + EFV	1	0	0	1	C
Tramadol	1	0	1	2	B
Triple therapy	0	0	1	1	C
Total	39^b^	14^b^	11^b^	64^b^ (377)	

^a^Supplemental drug ✓ given throughout the pregnancy

^b^Drugs other than Iron/folate; the number in parenthesis indicates the total number of drugs including iron/folate

#### FDA pregnancy risk categories

From a total of 377 drugs prescribed for pregnant women, 320 (84.88%) drugs were prescribed from FDA pregnancy risk category A; 33 (8.75%) drugs were from category B; 19 (5.04%) drugs were from category C and the rest 5 (1.33%) drugs were prescribed from category D. There was no drug prescribed from FDA pregnancy risk category X in this study (Table 3).

#### Dosage regimen related information

For supplemental drugs, only the drug name was stated and no other dosage regimen components specified. Upon investigating the components of dosage regimen for non-supplemental drugs, dosage form and strength were not recorded at all on medical records. Dose and frequency were written in almost all medical records (n = 56, 98.24%) followed by route of administration and duration of therapy, which were written on 48 (84.21%) and 23 (40.35%) patient medical records, respectively (Additional file 1: Figure S1).

### Discussion

Prescription medication use during pregnancy has become a common practice in many settings. Physicians prescribe and women use medicines with possibility of risks and without critically judging how harmful it may be for both mother and fetus [13–15]. In this study, the prevalence of drug use during pregnancy was found to be 85.1%. This value was comparable with the study conducted in northern Ethiopia [8] and Bahirdar city [12] where the prevalence of drug use during pregnancy was found to be 87.5% and 88.4%, respectively. With reference to this finding, lower and higher drug utilization were also reported in studies conducted at Ayder referral hospital, Mekele (62.2%) [16] and Nekemte town (96%) [17], respectively. Almost half of the drug used in the current finding was during the first trimester of pregnancy where the critical period of organogenesis lies and drug-induced teratogenicity is assumed to reach climax. Generally, drug use during pregnancy should be in compassionate manner and there must be vivid information justifying the potential benefit to the mother and the potential risk to the fetus.

Supplemental drug utilization accounted for 84.88% of total drug used, iron/folate utilization took 97.81% of supplemental drug therapy, which was by far higher than a study conducted in eight rural districts of Ethiopia where only 35.4% of pregnant women used iron supplements [18]. Study conducted in Addis Ababa [19] was somewhat in similar pattern of iron/folate utilization with the present study. The lower value observed in rural districts might be, in part, related to low health service coverage, distance from healthcare settings and awareness about the importance of such supplements for pregnant women.

Antibiotics were the most utilized medications from non-supplemental drugs in this study. This finding was found in concordant with previous studies conducted in HFSUH [20] and Adama referral hospital [1]. From toxicological perspectives, majority of antibiotics were from penicillins, cephalosporins and macrolids and all of which are actually from pregnancy risk category B where either animal reproductive studies have not demonstrated fetal risks but no controlled studies in pregnant women have been reported, or animal reproductive studies have shown an adverse effect that was not confirmed in controlled studies in women in the first trimester [21]. Maternal use of antibiotics during pregnancy has been associated with an increased risk of otitis media and ventilation tube insertions in the offspring [22]. During pregnancy, pharmacokinetic alterations in antibiotics require dose adjustment or careful monitoring and assessment [4, 23]. Antibiotic use during pregnancy has also shown a risk of spontaneous abortion and congenital malformations in the first trimester [24–27].

Looking at the overall distribution of drug utilization, majority of the drugs was from FDA category A (84.88%). This finding was in line with a study conducted in northern Ethiopia [8] where high number of drugs was used from this category. However, this finding had shown a gap from the study conducted in Sao Paulo, Brazil [28] and Fiche hospital, Ethiopia [29] in which only 20.55% and 20.83%, respectively, were from category A. The study conducted in a tertiary care hospital, India indicated that 91.13% of drugs used during pregnancy were from this category and was found slightly higher than the present finding [30]. In this study, 8.75% of drugs used were from FDA category B. This finding was in line with the study conducted in northern Ethiopia (7.9%) [8]. Higher prevalence of drug use was reported from studies conducted in Jimma [10] and western Nepal [31] where 48.7% and 60.2% of drugs were prescribed from FDA category B.

The use of category C drugs in this study was somewhat similar with the study conducted in urban heath center of Nanded where only 4.28% of drugs constituted this category [32]. In the present study, there were 5 (1.33%) drugs from FDA-category D and all of them were used for chronic illnesses. These drugs were the least utilized from FDA pregnancy risk category drugs which was in line with that of the study conducted in Brazil (1.85%) [28] and in Coastal town, India (3.13%) [33] where medications used for pregnant women were from this category. A study conducted in Addis Ababa [34] had a higher value than this finding whereas a study conducted in Ahmedabad, India revealed that there was no drug used from this category [35]. In category D, there is positive evidence of human fetal risk based on adverse reaction data from investigational or marketing experience or studies in humans, but potential benefits may warrant use of these drug in pregnant women despite potential risks [21]. In the current finding there was no drug used from FDA category X. This was similar with the study conducted in Adama [1], northern Ethiopia [8] and Banglore [36]. Studies indicated that there was a practice of prescribing drugs from category X in Omani (0.3%) [9], in Brazil (0.03%) [28], in Jimma, Ethiopia (7.4%) [10], in Bahirdar, Ethiopia (5.5%) [12], and in United States (4.6%) [11].

The FDA five letter system (A, B, C, D and X) of pregnancy risk category has been used since 1979. This system highlights toxicological consideration of drug use during pregnancy based on some evidences collected from preclinical and clinical trials. However, there are backlashes from customers to FDA forcing it to revise this letter system into new narrative system. While the new labeling improves the old format, it still does not provide a definitive “yes” or “no” answer in most cases. Clinical interpretation is still required on a case-by-case basis. The new system actually considers nursing mothers and males and females of reproductive potential [21].

### Conclusion

Most of the drugs utilized were supplemental in nature. There was no drug utilized by pregnant women from FDA pregnancy risk category X. Some drugs were utilized from FDA category D for life threatening chronic illnesses, despite the fact that some drugs might have risk for pregnant women and for which they have safe alternatives during pregnancy. There was insufficient drug information on patients’ medical records and some patient medical records didn’t have diagnosis for the prescribed drugs at all.

## Limitations

This study tried to address the overall drug utilization pattern during pregnancy. However, the study was not without limitations. This is a cross sectional study which couldn’t address the temporal relationship of drug exposure and pregnancy risk. Besides, the study utilized the old letter (A–X) system of FDA pregnancy risk categories which seem overly simplified despite the fact that the new narrative system has no cut-off point (requires critical judgment) and couldn’t be studied with retrospective chart review.

## Additional file

**Additional file 1: Figure S1.** Dosage regimen related information on medical records of pregnant women for non-supplemental drugs.

## Abbreviations

FDA: Food and Drug Administration
HFSUH: Hiwot Fana Specialized University Hospital
MCH: maternal and child health
