# Supplementary figures and images for: Drug utilization pattern among pregnant women attending maternal and child health clinic of tertiary hospital in eastern Ethiopia: Consideration of toxicological perspectives
Source: BMC Res Notes. 2018 Dec 4;11:858. doi: 10.1186/s13104-018-3966-5 (PMC6280540; doi:10.1186/s13104-018-3966-5)

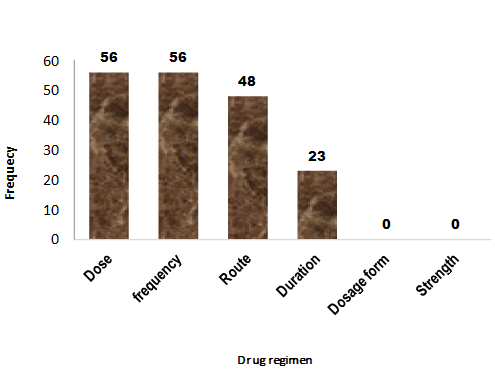

Supplement: Supplementary file 1 — Additional file 1: Figure S1. Dosage regimen related information on medical records of pregnant women for non-supplemental drugs. [file 13104_2018_3966_MOESM1_ESM.png]
